# SIRT1 inhibits adipogenesis and promotes myogenic differentiation in C3H10T1/2 pluripotent cells by regulating Wnt signaling

**DOI:** 10.1186/s13578-015-0055-5

**Published:** 2015-11-12

**Authors:** Yuanfei Zhou, Zheng Zhou, Wei Zhang, Xiaoming Hu, Hongkui Wei, Jian Peng, Siwen Jiang

**Affiliations:** Department of Animal Nutrition and Feed Science, College of Animal Science and Technology, Huazhong Agricultural University, Wuhan, 430070 People’s Republic of China; State Key Laboratory of Agricultural Microbiology, Division of Animal Infectious Disease, College of Veterinary Medicine, Huazhong Agricultural University, Wuhan, 430070 People’s Republic of China; The Cooperative Innovation Center for Sustainable Pig Production, Wuhan, 430070 People’s Republic of China; Key Laboratory of Swine Genetics and Breeding of Agricultural Ministry, and Key Laboratory of Agricultural Animal Genetics, Breeding and Reproduction of Ministry of Education, College of Animal Science and Technology, Huazhong Agricultural University, Wuhan, 430070 People’s Republic of China

**Keywords:** MSCs, Adipogenesis, Myogenic differentiation, SIRT1, Wnt signaling

## Abstract

**Background:**

The directed differentiation of mesenchymal stem cells (MSCs) is tightly controlled by a complex network. Wnt signaling pathways have an important function in controlling the fate of MSCs. However, the mechanism through which Wnt/β-catenin signaling is regulated in differentiation of MSCs remains unknown. SIRT1 plays an important role in the regulation of MSCs differentiation.

**Results:**

This study aimed to determine the effect of sirtuin 1 (SIRT1) on adipogenesis and myogenic differentiation of C3H10T1/2 cells. First, the MSC commitment and differentiation model was established by using 5-azacytidine. Using the established model, C3H10T1/2 cells were treated with SIRT1 activator/inhibitor during differentiation. The results showed that resveratrol inhibits adipogenic differentiation and improves myogenic differentiation, whereas nicotinamide promotes adipogenic differentiation. Notably, during commitment, resveratrol blocked adipocyte formation and promoted myotubes differentiation, whereas nicotinamide enhanced adipogenic potential of C3H10T1/2 cells. Furthermore, resveratrol elevated the expression of Cyclin D1 and β-catenin in the early stages. The luciferase assay showed that knockdown *SIRT1* inhibits Wnt/β-catenin signaling, while resveratrol treatment or overexpression *SIRT1* activates Wnt/β-catenin signaling. SIRT1 suppressed the expression of Wnt signaling antagonists *sFRP2* and *DACT1*. Knockdown *SIRT1* promoted adipogenic potential of C3H10T1/2 cells, whereas overexpression *SIRT1* inhibited adipogenic differentiation and promoted myogenic differentiation.

**Conclusions:**

Together, our results suggested that SIRT1 inhibits adipogenesis and stimulates myogenic differentiation by activating Wnt signaling.

## Background

Mesenchymal stem cells (MSCs) are multipotent stromal cells that can differentiate into adipocytes, myoblasts, osteoblasts and chondrocytes [1]. When triggered by appropriate condition, MSCs become committed to the adipocyte lineage. This process can be divided into two related stages: commitment and terminal differentiation [2, 3]. The differentiation into different cell lineages is determined by different factors and signaling pathways. Wnt signaling pathways have an important function in controlling the fate of MSCs [4]. Canonical β-catenin-dependent Wnt signaling maintains preadipocytes in an undifferentiated state through inhibition of the adipogenic transcription factors CCAAT/enhancer binding protein α (C/EBPα) and peroxisome proliferator-activated receptor γ (PPARγ) [5]. Canonical Wnt signalling has been implicated in satellite cell-related transdifferentiation and increasing myogenic potential [6]. Wnt signalling is influenced by potent antagonists, which exert their inhibitory effects at different points of the pathway. Although research has demonstrated that Wnt antagonists exert a crucial role during the differentiation process of preadipocytes into mature fat cells [2], research on the commitment process is lacking. Secreted frizzled-related proteins (sFRPs), extracellular antagonists of Wnt signaling, are induced during adipogenesis; constitutive overexpression of *sFRP1* in vitro promotes adipogenic differentiation through inhibition of canonical Wnt signaling [7]. Dacts, a homologue of Dapper, are antagonist to Wnt/β-catenin signaling. Knockdown of *Dact1* impairs adipogenesis and constitutive overexpression of *Dact1* promotes adipogenesis through inhibition/activation of the Wnt/β-catenin signaling [8]. However, the mechanism through which Wnt/β-catenin signaling is regulated in controlling the fate of MSCs remains unknown.

Sirtuin 1 (SIRT1) is a nicotinamide adenine dinucleotide-dependent lysine deacetylase that is involved in controlling the expression of key regulators of lifespan, cell defence, insulin secretion and adipocyte differentiation and metabolism [9–12]. The function of SIRT1 in adipogenesis has been reported by some researchers. Studies show that activation of SIRT1 by resveratrol blocks adipocyte development and increases the expression of osteoblast markers, whereas inhibition of SIRT1 by nicotinamide increases adipocyte number and expression of adipocyte markers in C3H10T1/2 [13]. In addition, resveratrol regulates cell cycle exit and induces C2C12 cells differentiation, controls muscle-specific proteins synthesis [14]. However, the mechanisms of SIRT1 in regulating adipogenesis and myogenic differentiation remain unclear. We hypothesize that SIRT1 controls adipogenesis and myogenesis of MSCs by regulating the Wnt signaling pathway.

C3H10T1/2 stem cell line was originally isolated from C3H mouse embryos. It behaves in a manner similar to that of MSCs, making C3H10T1/2 cells a useful MSC model [15]. Previous research has shown that low concentrations of the DNA methylation inhibitor 5-azacytidine (5-AZA) converts C3H10T1/2 cells into differentiated chondrocytes, adipocytes and skeletal muscles [16]. 5-AZA is an effective agent for determining the fate of MSCs. In the present study, we aimed to investigate the function of SIRT1 in regulating MSC commitment into the adipogenic lineage. Our findings may potentially have a role in fat formation and lead to the development of novel therapeutic approaches to human obesity-related diseases.

## Results

### Establishment of the MSC commitment and differentiation model

**Fig. 1:**
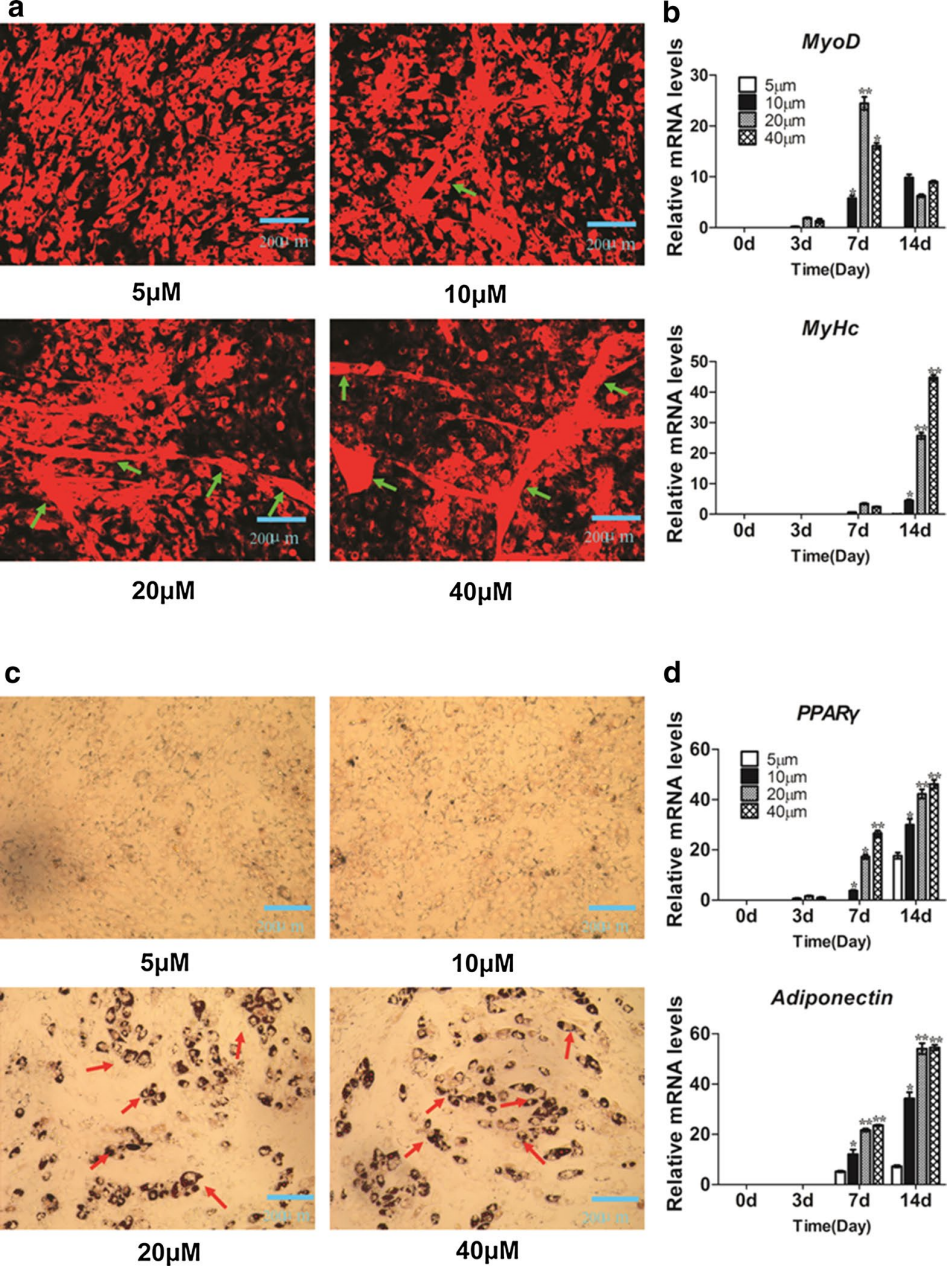
5-Azacytidine induces C3H10T1/2 cells into differentiated adipocytes and myocytes. C3H10T1/2 cells were treated with 5, 10, 20, and 40 μM 5-azacytidine for 3 days, then changed GM culture treated for next 14 days. **a** Cells were fixed and stained with DiI on day 14 days after GM Culture. **b** The relative expression profile of myogenic marker genes: *MyoD* and *MyHc* were determined using Real-time PCR at 0, 3, 7, and 14 days after treatment. **c** Oil Red-O staining of cells on day 14 days after GM culture. **d** The relative expression profile of adipogenic marker genes: *PPARγ* and *adiponentin* were determined using Real-time PCR at 0, 3, 7, and 14 days after treatment. The *green arrow* points to the myotube. The *red arrow* points to the lipid droplet. Data represent mean ± SEM from three independent experiments. *P < 0.05; **P < 0.01

To establish a commitment and differentiation model of MSCs, C3H10T1/2 cells were induced with 5, 10, 20 or 40 µM of 5-AZA for 3 days and cultured in GM for another 14 days. Myogenic and adipogenic phenotypes were measured by using DiI staining and Oil Red O staining, respectively. 5-AZA treatment increased myocyte formation (Fig. 1a) and lipid accumulation (Fig. 1c) with the concentration gradient. Treatment with 20 and 40 µM 5-AZA effectively induced myocyte and adipocyte formation. Cells cultured in GM were harvested at 0, 3, 7 and 14 days to determine the mRNA expression profile of marker genes for myogenesis and adipogenesis. The results showed that the myogenic marker gene *MyoD* was highly expressed at 7 days of inducing differentiation, and the late differentiation marker *MyHc* was highly expressed at 14 days of differentiation (Fig. 1b). Meanwhile, the adipogenic markers PPARγ and adiponectin were highly expressed at 7 and 14 days of differentiation, respectively (Fig. 1d). The myogenic or adipogenic marker gene expressions of 20 and 40 µM 5-AZA-treated groups were the highest. The values were significantly higher than the 5 and 10 µM recorded for 5-AZA-treated cells. The results indicated that 20 and 40 µM 5-AZA treatment effectively induced MSC commitment to the myocyte and adipocyte lineage. Thus, treatment with 20 µM 5-AZA was chosen to be used for subsequent study.

**Fig. 2:**
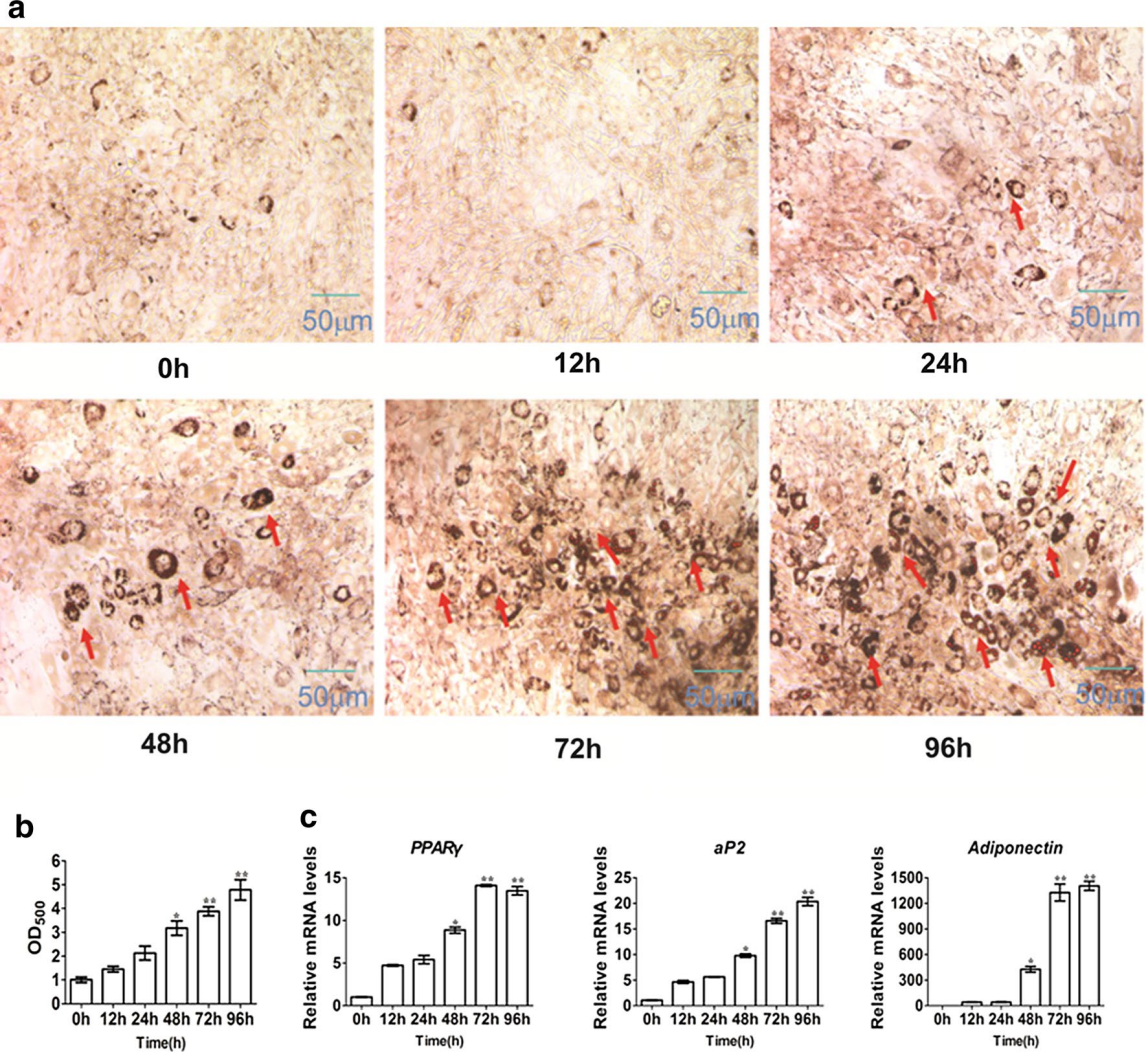
5-Azacytidine induces C3H10T1/2 cells into differentiated adipocytes for different treatment time. The C3H10T1/2 cells were induced with 20 μM 5-azacytidine for 0, 12, 24, 48, 72, and 96 h, then changed adipocyte differentiation medium for 8 days. **a** After 8 days of culture in adipocyte differentiation medium, the cells were stained with Oil Red-O. **b** The quantification of **a**. The Oil-Red O stain was extracted with 100 % isopropanol and the optical density at 500 nm (OD_500_) was determined. **c** Real-time PCR analyzed *PPARγ*, *aP2* and *adiponentin* at 8 days after adipogenic medium stimulation. The *red arrow* points to the lipid droplet. Data represent mean ± SEM from three independent experiments. *P < 0.05; **P < 0.01

Using 20 µM 5-AZA treatment, C3H10T1/2 cells were induced for 0, 12, 24, 48, 72 and 96 h. After 8 days of culture in pre-adipocyte adipogenic cocktail, the cells were stained with Oil Red O to measure adipogenic phenotype. The results showed that no adipocyte was formed in groups treated with 5-AZA for 0, 12 and 24 h. Adipocytes were formed by treatment with 5-AZA for more than 48 h, and adipocyte number increased with treatment time (Fig. 2a, b). Through real-time polymerase chain reaction (PCR) analyses, we determined that 5-AZA significantly induced the mRNA expression of adipogenic markers (*PPARγ*, *aP2*, and *adiponectin*) with the increase in treatment duration (Fig. 2c). In summary, cells treated with 20 µM 5-AZA for 3 days effectively induced the conversion of MSCs to adipocytes, and will be adopted for subsequent study.

### Effect of SIRT1 on MSC adipogenic differentiation

**Fig. 3:**
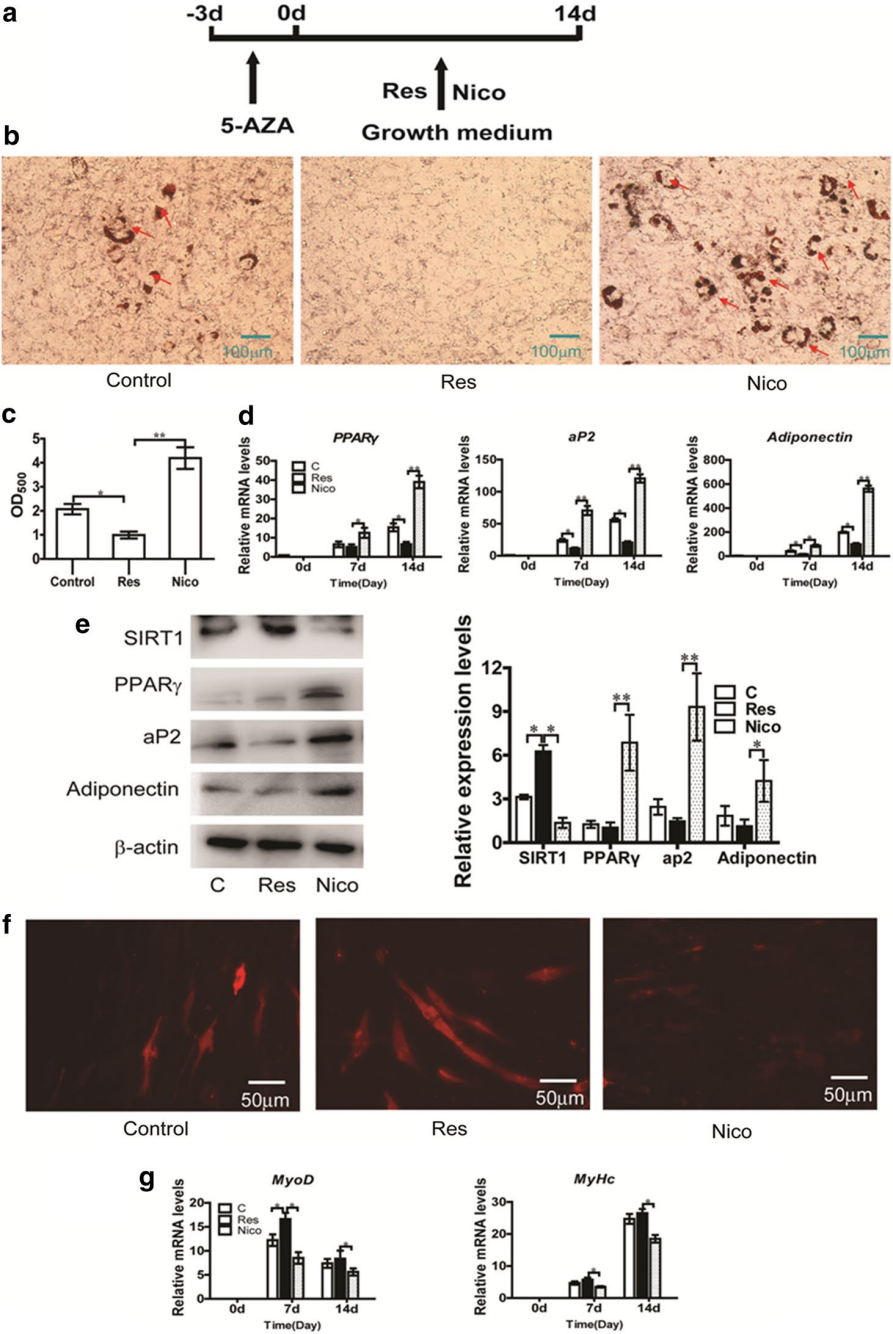
Effect of SIRT1 activation/inhibition on myogenic differentiation and adipogenic differentiation of MSCs. **a** Schematic representation of the experimental design: C3H10T1/2 cells were treated with 20 μM 5-azacytidine for 3 days, then SIRT1 activator (50 μM resveratrol)/inhibitor (10 mM nicotinamide) was added to GM of C3H10T1/2 cells for 14 days. **b** Cells were fixed and stained with Oil Red-O on day 14 days after GM culture. **c** The quantification of **b**. The Oil-Red O stain was extracted with 100 % isopropanol and the optical density at 500 nm (OD_500_) was determined. **d** The relative expression profile of adipogenic marker genes: *PPARγ*, *aP2* and *adiponentin* were determined using Real-time PCR. **e** PPARγ, aP2 and adiponentin protein expression detected by western blotting (*left*) and densitometry analysis (*right*) on day 14 days after GM culture. **f** Cells were fixed and stained with Dil on day 14 days after GM culture. **g** The relative expression profile of myogenic marker genes: *MyoD* and *MyHc* were determined using Real-time PCR. The *red arrow* points to the lipid droplet. Data represent mean ± SEM from three independent experiments. *P < 0.05; **P < 0.01

To explore the role of SIRT1 in adipogenic differentiation of MSCs, SIRT1 activator (resveratrol)/inhibitor (nicotinamide) was added to GM of C3H10T1/2 cells after 5-AZA treatment during differentiation, as shown in Fig. 3a. First, we explored the effect of SIRT1 on C3H10T1/2 cell differentiation using Oil Red O staining for lipid droplets. Oil Red O staining showed that compared with the control group, resveratrol inhibited the adipogenic differentiation of C3H10T1/2, whereas nicotinamide increased the adipogenic differentiation of C3H10T1/2 (Fig. 3b, c). The mRNA and protein levels of the adipogenic markers (*PPARγ*, *ap2* and *adiponectin*) were also slightly reduced in the resveratrol group (Fig. 3d, e). By contrast, the mRNA and protein levels of the adipogenic marker genes significantly improved in the nicotinamide group (Fig. 3d, e). The results suggested that activation of SIRT1 by resveratrol inhibits the adipogenic differentiation of C3H10T1/2, whereas inhibition of SIRT1 by nicotinamide promotes the adipogenic differentiation of C3H10T1/2. Dil staining showed that resveratrol promoted myotubes formation, whereas nicotinamide increased the myogenic differentiation of C3H10T1/2 (Fig. 3f). We next examined the mRNA levels of myogenic markers *MyoD* and *MyHc* during differentiation (Fig. 3g), suggesting that resveratrol significantly increased myogenic differentiation.

### Effect of SIRT1 on MSC adipogenic commitment

**Fig. 4:**
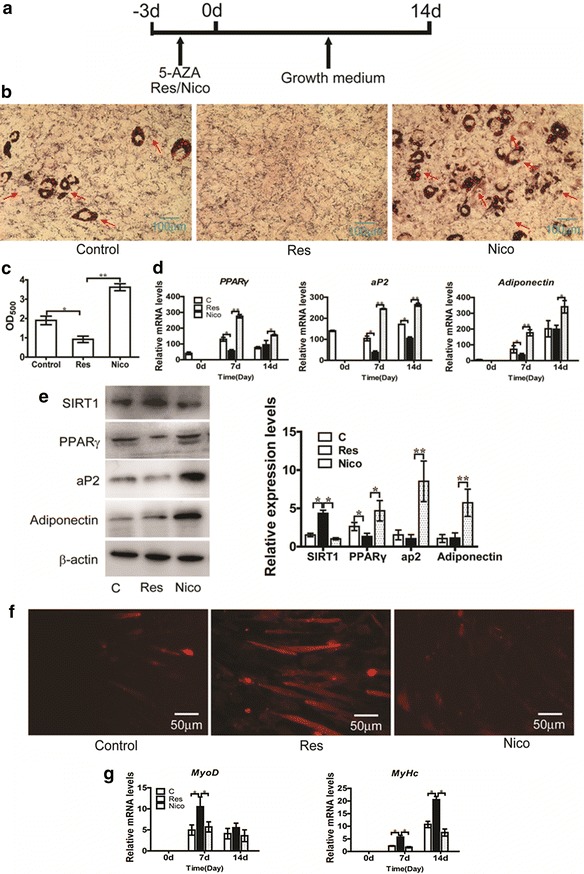
Effect of SIRT1 activation/inhibition on myogenic commitment and adipogenic commitment of MSCs. **a** Schematic representation of the experimental design: 50 μM resveratrol or 10 mM nicotinamide was added to C3H10T1/2 cells with 20 μM 5-AZA during commitment for 3 days, then C3H10T1/2 cell was cultured in GM for 14 days. **b** Cells were fixed and stained with Oil Red-O on day 14 days after GM culture. **c** The quantification of **b**. The Oil-Red O stain was extracted with 100 % isopropanol and the optical density at 500 nm (OD_500_) was determined. **d** The relative expression profile of adipogenic marker genes: *PPARγ*, *aP2* and *adiponentin* were determined using Real-time PCR. **e** PPARγ, aP2 and adiponentin protein expression detected by western blotting (*left*) and densitometry analysis (*right*) on day 14 days after GM culture. **f** Cells were fixed and stained with Dil on day 14 days after GM culture. **g** The relative expression profile of myogenic marker genes: *MyoD* and *MyHc* were determined using Real-time PCR. The *red arrow* points to the lipid droplet. Data represent mean ± SEM from three independent experiments. *P < 0.05; **P < 0.01

Using the established model, MSCs were treated with SIRT1 activator/inhibitor during commitment to study the effect of SIRT1. To explore the function of SIRT1 in C3H10T1/2 committed to the adipocyte lineage, SIRT1 activator (resveratrol)/inhibitor (nicotinamide) was added to C3H10T1/2 cells with 5-AZA during commitment, as shown in Fig. 4a. As shown in Fig. 4b and c, Oil Red O staining demonstrated results similar to those of differentiation. Compared with the control group, resveratrol attenuated C3H10T1/2 cells committed to the adipocyte lineage, whereas nicotinamide increased C3H10T1/2 committed to the adipocyte lineage. The mRNA and protein levels of the adipogenic markers were also slightly suppressed in the resveratrol group (Fig. 4d, e). By contrast, mRNA and protein levels of the adipogenic marker genes were significantly enhanced in the nicotinamide group (Fig. 4d, e). Taken together, our data indicated that activation of SIRT1 by resveratrol inhibits C3H10T2/1 adipogenic commitment, whereas inhibition of SIRT1 by nicotinamide promotes C3H10T2/1 adipogenic commitment. Dil staining demonstrated results similar to those of differentiation, resveratrol enhanced the number of myotubes compared with the control group (Fig. 4f). The *MyoD* and *MyHc* mRNA levels were determined via real-time PCR (Fig. 4g). The results showed that resveratrol significantly increased myogenic commitment and differentiation.

### Regulation of Wnt signaling by SIRT1 during MSC adipogenic commitment

**Fig. 5:**
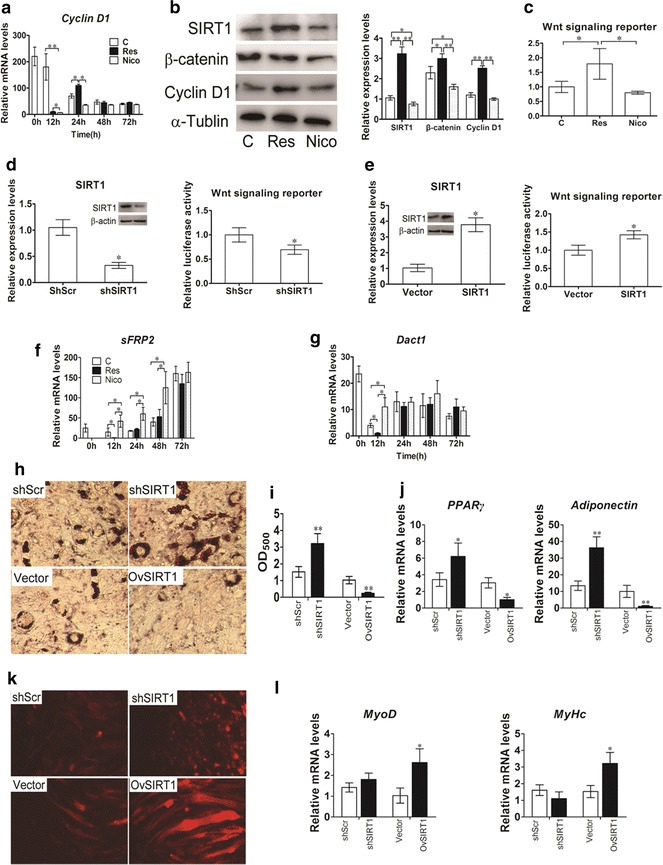
Effect of SIRT1 activation/inhibition on Wnt signaling pathways during commitment of MSCs. 50 μM resveratrol or 10 mM nicotinamide was added to C3H10T1/2 cells with 20 μM 5-AZA during commitment for 3 days. **a** The relative expression profiles of *CyclinD1* mRNA was determined using Realtime-PCR. **b** SIRT1, β-catenin and Cyclin D1 protein expression detected by western blotting at 24 h. **c** The luciferase reporter activity was measured at 24 h after transfection with resveratrol or nicotinamide. **d** RNAi *SIRT1* plasmid was co-transfected into C3H10T1/2 cells with TCF-receptor. **e** Overexpression *SIRT1* plasmid was co-transfected into C3H10T1/2 cells with TCF-receptor. The luciferase reporter activity was measured 48 h after transfection. **f** *sFRP2* mRNA measured by Realtime-PCR and **g** *Dact1* mRNA measured by Realtime-PCR at 0, 12, 24, 48, and 72 h. **h** Cells were fixed and stained with Oil Red-O on day 14 days after GM culture. **i** The quantification of **h**. The Oil-Red O stain was extracted with 100 % isopropanol and the optical density at 500 nm (OD_500_) was determined. **j** The relative expression profile of adipogenic marker genes: *PPARγ*, *adiponentin* were determined using Real-time PCR. **k** Cells were fixed and stained with Dil on day 14 days after GM culture. **l** The relative expression profile of myogenic marker genes: *MyoD* and *MyHc* were determined using Real-time PCR. Data represent mean ± SEM from three independent experiments. *P < 0.05; **P < 0.01

Using the established model, C3H10T1/2 cells were treated with SIRT1 activator/inhibitor during commitment and differentiation. Cells were harvested at 0, 12, 24, 48 and 72 h of commitment. The mRNA expression profile of the Wnt signaling pathway target gene (*Cyclin D1*) and Wnt antagonists (*DACT1* and *sFRP2*) were determined using real-time PCR. The protein expression profiles of Cyclin D1 and β-catenin, a key component of the Wnt signaling pathway, were determined through Western blot. The results showed that compared with the control group, *Cyclin D1* mRNA expression of the resveratrol-treated group was significantly higher at 24 h of commitment, whereas *Cyclin D1* expression of the nicotinamide-treated group was significantly lower than that of the control group at 24 h of commitment. Resveratrol promotes the expression of SIRT1, whereas nicotinamide inhibits the expression of SIRT1 at 24 h of commitment (Fig. 5b). Meanwhile, SIRT1 inhibition reduces the expression of β-catenin and Cyclin D1 protein (Fig. 5b). To further investigate whether SIRT1 mediated Wnt/β-catenin signaling, we used the TCF-reporter of Wnt/β-catenin signaling. The luciferase assay showed that resveratrol increased Wnt/β-catenin signaling (Fig. 5c). RNAi expression of *SIRT1* suppressed the activity of TCF-reporter (Fig. 5d), whereas overexpression of *SIRT1* significantly increased the luciferase activity in C3H10T1/2 cells (Fig. 5e). The results indicated that SIRT1 controls MSC commitment by relegating Wnt signaling. Furthermore, we explore the affects of SIRT1 on Wnt signaling antagonists. The results showed that resveratrol significantly decreased *sFRP2* and *DACT1* expression at 24 h, whereas nicotinamide significantly increased the expression of *sFRP2* and *DACT1* during commitment (Fig. 5f, g). Furthermore, RNAi *SIRT1* increased the formation of lipid drops, whereas overexpression *SIRT1* reduced the formation of lipid drops (Fig. 5h, i). The mRNA expression of *PPARγ* and *adiponectin* were similar to the phenotype (Fig. 5j). RNAi *SIRT1* blocked myogenic differentiation, whereas overexpression *SIRT1* promoted myogenic differentiation of C3H10T1/2 cell (Fig. 5k, l). The results suggested that during commitment, SIRT1 affects Wnt signaling may via regulating Wnt signaling antagonists expression.

## Discussion

We established a suitable model for investigating MSC commitment and differentiation. Our data suggested that SIRT1 is a negative regulator of MSC commitment and differentiation to adipocytes. During MSC commitment, SIRT1 affects Wnt signaling by regulating Wnt signaling antagonists *sFRP2* and *DACT1* expression and determining stem cell fate.

Previous research has shown that low concentrations of 5-AZA convert C3H10T1/2 cells into differentiated chondrocytes, adipocytes and skeletal muscle [16]. W Wakitani et al. demonstrated that 5-AZA in culture medium induced bone marrow MSCs to differentiate into myogenic cells and adipocytes [17]. Treatment with 5-AZA affected MSC commitment in a dose-dependent manner [17], which is consistent with our results. We determined that the relatively high concentrations of 5-AZA treatment (20 and 40 µM) effectively induced MSC commitment to the myocyte and adipocyte lineage. The relatively lower concentration of 5-AZA (20 µM) was used for our study.

SIRT1 has an important function in a wide variety of processes, including cell proliferation and differentiation [18], apoptosis [19] and metabolism [20]. Picard et al. first reported that SIRT1 represses PPARγ by docking with its co-factors NCoR and SMRT in 3T3-L1 pre-adipocytes, which impairs adipogenic differentiation [12]. Previous studies have indicated that SIRT1 has a key modulatory role in animal fat metabolism and muscle development [21, 22]. In this study, we demonstrated that activation of SIRT1 by resveratrol inhibits adipogenic differentiation, whereas inhibition of SIRT1 by nicotinamide promotes adipogenic differentiation in an established model of MSCs induced by 5-AZA (Fig. 3), consistent with previous study. Resveratrol inhibits human pre-adipocyte proliferation and adipogenic differentiation in an SIRT1-dependent manner [23]. Conversely, nicotinamide significantly induces the differentiation of pre-adipocyte into adipocyte [24]. In addition, studies have suggested that SIRT1 promotes osteogenesis and decreases adipogenesis of MSCs [25–27]. Resveratrol/nicotinamide was added to C3H10T1/2 cells with 5-AZA during commitment. Notably, our data showed that resveratrol inhibits adipogenic commitment and differentiation and promotes myogenic differentiation (Fig. 4). By contrast, nicotinamide promotes adipogenic commitment and differentiation (Fig. 4). Activation of SIRT1 by consistently adding resveratrol blocked adipocyte development and increased the expression of osteoblast markers in C3H10T1/2 [1]. However, this finding did not justify the role of SIRT1 during commitment of MSCs. Similarly, activation of SIRT1 activity with resveratrol for the duration increased muscle precursor cell proliferation, whereas inhibition of SIRT1 with nicotinamide lowered proliferation [27, 28]. Our finding provides further evidence that SIRT1 is a negative regulator of MSC differentiation to adipocyte. More interestingly, similar to the differentiation stage, SIRT1 suppresses adipocyte lineage commitment of MSCs.

Wnt signaling pathways have important functions in controlling the fate of MSCs [4]. Wnt/β-catenin signaling promotes the differentiation of MSCs into myocytes and osteocytes and suppresses commitment to the adipocyte lineage [4]. However, the molecular mechanism of SIRT1 regulation by Wnt signaling is unclear. Our study demonstrated that resveratrol treatment led to an increased mRNA level of *Cyclin D1*, a well-known Wnt signaling target gene. In addition, resveratrol treatment more significantly increased β-catenin and Cyclin D1 protein expression compared with nicotinamide treatment (Fig. 5). Inhibition SIRT1 reduced the activity of TCF-reporter, whereas activation SIRT1 significantly increased the luciferase activity in C3H10T1/2 cells (Fig. 5). Zhou et al. showed that resveratrol treatment was significantly higher in cells transfected with TOPFlash reporter vector than in cells transfected with the negative control FOPFlash reporter vector [29]. In addition, their results suggested that an increase in the level of β-catenin in response to resveratrol is mediated by a downregulation of the kinase activity of GSK-3β [29]. However, SIRT1 can deacetylate histones and a number of non-histone substrates. Researchers have proposed that SIRT1 can regulate the Wnt signaling pathway through other means. Simic et al. showed that SIRT1 deacetylates β-catenin to promote its accumulation in the nucleus leading to transcription of genes for MSC differentiation [30]. sFRPs are extracellular Wnt signaling antagonists that directly bind Wnt molecules and sequester them from their membrane-bound receptors [2]. Park et al. found that sFRP levels increased gradually during adipogenesis via inhibition of Wnt signaling in human amniotic MSCs [31]. Our study suggested that resveratrol treatment significantly increased the mRNA level of sFRP2, whereas nicotinamide treatment significantly suppressed the mRNA level of sFRP2 in the early stages of commitment of MSCs. SIRT1 localises to the promoter of sFRP2, directly contributing to the aberrant epigenetic silencing of breast cancer cells [32, 33]. Dacts are intracellular mediators of Wnt signaling that interact with the protein Dishevelled, thereby inhibiting conduction of the signal from the Fz/LRP receptor complex [2]. Our study suggested that resveratrol treatment significantly increased the mRNA level of Dact1, whereas nicotinamide treatment significantly suppressed the mRNA level of Dact1 in the early stages of commitment of MSCs. Previous studies have shown that Dact1 regulates adipogenesis through coordinated effects on gene expression that selectively alter intracellular and paracrine/autocrine components of the Wnt/β-catenin signaling pathway [8]. Together with our findings, these data suggest that SIRT1 may block adipogenesis of MSCs by inhibiting the expression of the Wnt signaling antagonists *sFRP2* and *DACT1*. Future studies will focus on the molecular mechanisms through which SIRT1 regulates Wnt signaling during adipogenesis of MSCs.

In conclusion, although further studies are required to elucidate the molecular mechanisms underlying SIRT1-mediated adipogenesis and myogenic differentiation of MSCs, these results clearly identify SIRT1 as a negative regulator of MSC commitment and differentiation to adipocyte, and as a positive regulator for myogenic differentiation in MSCs. SIRT1 may affect MSC fate by regulating Wnt signaling pathway. These findings further confirmed that SIRT1 may potentially have a role in the development of obesity-related diseases.

## Methods

### Cell culture

C3H10T1/2 cell was purchased from Cell Bank of Type Culture Collection of China Science Academy (Shanghai, China) and grown in Dulbecco’s modified Eagle’s medium (DMEM) supplemented with 10 % fetal bovine serum (FBS) (Gibco BRL) in a 5 % CO_2_ incubator at 37 °C. To induce adipocyte lineage commitment, C3H10T1/2 stem cells were plated at low density of 40–50 % and cultured in DMEM containing 10 % calf serum with 5-AZA (A3656, St. Louis, MO, USA). After the cells reached postconfluence, they were maintained by using the growth medium (GM). For adipocyte differentiation assay, cells were induced with a cocktail of dexamethasone (1 μM), insulin (10 μg/ml), isobutylmethyxanthine (0.5 mM) (DMI) and 10 % FBS. 2 days after induction, cells were maintained in DMEM containing insulin (10 μg/ml) for an additional 2 days and 10 % FBS until they were ready for harvest [34].

### DiI staining and Oil red-O staining

After treatment with GM for 14d, C3H10T1/2 cells were stained with the membrane probe DiI (Beyotime, Haimen, China) at 37 °C for 5–10 min, fixed with 4 % paraformaldehyde for 15 min. The cells were mounted in mounting reagent (DTT/PBS/glycerol). Accumulation of triglyceride content in differentiated cells was visualized by staining with Oil red-O (Sigma-Aldrich). Cells were washed twice with PBS and fixed with 10 % formaldehyde for 45 min at room temperature. After washing with distilled water twice and 50 % isopropanol once, the cells were stained for 1 h at room temperature with filtered Oil red O/60 % isopropanol solution. The cells were washed twice with distilled water and twice with PBS. Adipocytes stained red were recorded by light microscopy (Leica German). To quantify staining, Oil Red O was extracted from the cells with 100 % isopropanol, and the optical density was measured at 500 nm (OD_500_).

### Real-time quantitative PCR

Total RNA was isolated from cells using Trizol reagent following the protocol provided by the manufacturer (Invitrogen) and reverse transcribed according to the manufacturer’s protocol (Takara, Dalian, China). Real-time PCR was performed by mixing cDNA with primers, and iTaq™ Universal SYBR^®^ Green Supermix quantitative PCR analysis reactions (Bio-Rad, USA). Real-time PCR was performed using a LightCycler^®^ 480 System with supplied software (Roche, USA), according to the manufacturer’s instructions. RNA expression levels were compared after normalization to endogenous β-actin. The primer sequences used in this study are listed in Table 1.

**Table 1:** Primer sequences in this study

Gene	Primers (sense/antisense 5′–3′	Product length (bp)	Ta (°C)
*MyoD*	CGCTCCAACTGCTCTGATGGCAT	280	61
	GGGTTCCCTGTTCTGTGTCGCTT		
*MyHc*	TCGGGGTCTTGGACATTGCTGG	342	61
	CTTTGGCGGGTTTGGGCTTCTG		
*PPARγ*	TGGGTGAAACTCTGGGAGATTC	250	60
	AGAGGTCCACAGAGCTGATTCC		
*aP2*	GTGTGATGCCTTTGTGGGAAC	235	60
	CCTGTCGTCTGCGGTGATT		
*adiponentin*	GCTCTCCTGTTCCTCTTAATCCT	437	60
	CCAGTGCTGCCGTCATAATG		
*Cyclin D1*	AAATGCCAGAGGCGGATGAG	200	60
	AAGAAAGTGCGTTGTGCGGT		
*sFRP2*	AATCGGCATCTAAGTCTT	129	56
	GCAATGAGGAATGGTTAC		
*DACT1*	TCTGAGGAATGGAAGTGTG	169	58
	GTCTGTCTTTGAGTCTTTGG		
*β-actin*	GGCACCACACCTTCTACAATG	133	60
	GGGGTGTTGAAGGTCTCAAAC		

### Western blotting

Western blotting was performed as previously described. Whole-cell protein lysates were extracted with a solution containing 20 mM Tris–HCl (pH 7.5), 150 mM NaCl, 1 % Triton X-100, 10 mM Na_4_P_2_O_7_, 1 mM Na_3_VO_4_, 2 mM EDTA, 0.5 mM leupeptin, and 1 mM PMSF (Beyotime, China). The protein concentrations were determined using a Bradford Protein Assay Kit (Beyotime, China), and proteins were separated by 10 % SDS-PAGE. The separated proteins were then transferred to polyvinylidene difluoride (PVDF) membranes (Bio-Rad, USA). Membrane blocking to prevent nonspecific binding was done with TTBS buffer [10 mM Tris–HCl (pH 7.6), 150 mM NaCl, 0.1 % Tween 20] containing 5 % skim milk powder. The blocked membranes were then incubated with a mouse specific anti-SIRT1 antibody (number 2028; Cell Signaling Technology Inc, Danvers, MA, USA), or a rabbit polyclonal anti-PPARγ (number sc-1984; Santa Cruz), or a rabbit polyclonal anti-FABP4 (number 2120; Cell Signaling Technology Inc.), or a rabbit monoclonal anti-adiponectin (number 2789; Cell Signaling Technology Inc.), α-Tubulin (number sc-53646; Santa Cruz), and β-actin (number 3700; Cell Signaling Technology Inc.) for overnight at 4 °C. Secondary antibodies were used according to the manufacturer’s instructions. Secondary-antibody binding was detected using an enhanced chemiluminescence detection kit (Thermo Fisher Scientific, USA) according to the manufacturer’s instructions. Protein levels were normalized to β-actin using Image J analysis software.

### Transient transfection assays

For transient transfection assays, C3H10T1/2 cells were seeded to 24-well plate at 5 × 10^4^ cells/well 24 h before transfection. The cells were transiently transfected with plasmids at 80 % confluence using Lipofectamine 2000 reagent (Invitrogen) according to the manufacturer’s instructions. The DNA/reagent ratio was 1 μg/2 μL. After 24 h transfection, the cells were harvested for subsequent analysis. The RNA interfering (RNAi) plasmid pSIREN-RetroQ-ZsGreen (pSIREN-SIRT1) (siSIRT1) 5′-GATGAAGTTGACCTCCTCA-3′, synthesized according to the literature (Picard et al. [12]). pCDNA 3.1-SIRT1 plasmid was kindly provided from Dr. Zhai (Chinese Academy of Sciences).

### Luciferase reporter assay

The TOPflash plasmid (Millipore) was used to monitor the Wnt/β-catenin signaling. The TOP/FOP Flash assays were performed according to the manufacturer’s instructions. The cells were treated as indicated, and luciferase activity was measured with the Dual-Luciferase reporter assay system (Promega).

### Statistical analyses

Results are presented as the means ± standard errors. Statistical analysis was performed with SAS. Version 8 (SAS Inc., Chicago, IL, USA). Data were analyzed by Duncan’s multiple-range test was performed if differences were identified among the groups at P < 0.05.

## Abbreviations

MSCs: mesenchymal stem cell
sFRP: secreted frizzled-related protein
SIRT: sirtuin
5-AZA: 5-azacytidine
FBS: fetal bovine serum
GM: growth medium
